# Is latent *Toxoplasma gondii* infection associated with the occurrence of schizophrenia? A case-control study

**DOI:** 10.1371/journal.pone.0270377

**Published:** 2022-06-23

**Authors:** Muluneh Ademe, Tadesse Kebede, Solomon Teferra, Melkam Alemayehu, Friehiwot Girma, Tamrat Abebe

**Affiliations:** 1 Department of Microbiology, Immunology and Parasitology, College of Health Sciences, Addis Ababa University, Addis Ababa, Ethiopia; 2 Department of Psychiatry, School of Medicine, College of Health Sciences, Addis Ababa University, Addis Ababa, Ethiopia; 3 Department of Pediatrics and Child Health Nursing, School of Health Sciences, College of Medicine and Health Sciences, Bahir Dar University, Bahir Dar, Ethiopia; Abadan University of Medical Sciences, ISLAMIC REPUBLIC OF IRAN

## Abstract

**Introduction:**

Neurotropic pathogens such as *Toxoplasma gondii (T*. *gondii)* which result in chronic infections in the brain are associated with mental illnesses. In view of this, a growing body of literature has revealed the possible interaction of schizophrenia and *T*. *gondii* infection.

**Method:**

A case-control study was conducted from February 2018 to January 2019 among 47 Schizophrenia patients and 47 age and sex-matched controls. Data was collected using a structured questionnaire. Serum was used for serological analysis of anti-*T*. *gondii* IgG and IgM antibodies through chemiluminescent immunoassay. Proportions and mean with standard deviations (SD) were used as descriptive measures and variables with p-values <0.05 were considered as statistically significant and independently associated with schizophrenia.

**Result:**

The mean ages of schizophrenia patients and controls were 29.64 ± 5.8 yrs and 30.98 ± 7.3 yrs, respectively. We found that 81.9% (77/94) of the study subjects had a positive anti-*T*. *gondii* IgG antibody. While the difference is statistically insignificant, schizophrenic patients have a marginally higher seroprevalence of toxoplasmosis than controls (87.2% vs 80.9%; p = 0.398). Schizophrenia cases who live in homes with soil floors have a significantly higher *T*. *gondii* infection as compared to those who live in homes with cement/ceramic floors (90.9% vs 33.3%; p = 0.004). Furthermore, there was a significantly lower *T*. *gondii* infection among schizophrenic cases who were taking antipsychotic medication for more than three yrs (79.3% vs 100.0%, p = 0.039). On the other hand, among all study subjects who have *T*. *gondii* infection, subjects who are addicted to khat and alcohol were about seven times more likely to develop schizophrenia (71.4% vs 47.7%, OR = 7.13, p = 0.024).

**Conclusion:**

Our data is not sufficient to show a significant positive correlation between *T*. *gondii* infection and schizophrenia. For study subjects with *T*. *gondii* infection, addiction to khat and alcohol is one of the risk factors for schizophrenia.

## Introduction

Toxoplasmosis is an intracellular parasitic disease that is acquired mainly through the ingestion of water or food which is contaminated with *Toxoplasma gondii (T*. *gondii*) oocysts. The major sources of *T*. *gondii* infection are infected cat feces and undercooked meat containing tissue cysts. Usually, primary *T*. *gondii* infection is subclinical [1]. *T*. *gondii* parasites form resting cysts (bradyzoites), particularly in the muscle and brain. *T*. *gondii* results in a latent infection that lasts a lifetime. The tissue cysts of this parasite are resistant to virtually all available medications [2,3]. Chronic toxoplasmosis is estimated to affect over a third of humans worldwide [4]. To date, the abilities of neurotropic pathogens, including Herpesviridae [5], Bornavirus [6], Chlamydia [7], and *T*. *gondii* [8] to establish chronic infections within the brain tissue have been associated with the onset of different mental illnesses.

The implication of the possible interaction of *T*. *gondii* infection and schizophrenia dated over the past half-century [9]. Since then, a growing body of literature, as reviewed in [8,10,11], has studied the interaction of schizophrenia and *T*. *gondii* infection which is marked by an increased anti-*T*. *gondii* IgG antibody. Yet, the exact mechanism of interaction between the two is not clearly understood. However, based on the available evidence, the brain damage which is attributed to the local inflammatory reaction in response to resting tissue cysts in the brain, on the one hand, is linked to the onset of schizophrenia and other psychoses [12,13]. Contrarily, toxoplasmosis affects the levels of dopamine, norepinephrine, and other neurotransmitters in which their overproduction will result in necrotizing brain lesions [14,15]. In view of this, an increase in the levels of dopamine, due to either host nitric oxide-mediated dopamine release or *T*. *gondii*-mediated tyrosine hydroxylases (mostly studied in rodents), observed in the brain of chronic *Toxoplasma*-infected hosts has been linked to the possible association of latent (chronic) toxoplasmosis and schizophrenia [14,16,17].

So far, the roles of latent *T*. *gondii* infection in manipulating the host’s behavior have been well shown in different animal and human studies. Berdoy and colleagues, in an experimental model, have demonstrated that a rat infected with *T*. *gondii* has a reduced natural aversion to the odor of felines which is usually called the “fatal attraction phenomenon” [18]. Such behavioral changes in *Toxoplasma*-infected rodents are suggested to enhance the transmission of the parasite. A similar study by Flegr *et al*. on *Toxoplasma*-infected humans came up with an impressive finding in which *Toxoplasma*-infected men had an increased attractiveness to cat odor [19]. Besides, humans with *T*. *gondii* infection have been associated with a reduced novelty seeking score in Cloninger’s temperament and character inventory (TCI) test [20,21], an increased risk of suicides [22,23], and a changed super-ego strength, pretension, and affectothymia [24,25]. Owing to their roles in manipulating the host behavior, latent *T*. *gondii* infections have been considered as one potential parasitic infection to influence the host’s mental health [18].

In areas where there is a high burden of *T*. *gondii* infection, such as France and Ireland, high admission rates for schizophrenia have been reported [14]. In Ethiopia, where this study was conducted, there is a high infection rate of toxoplasmosis which ranges from 18.5% to 96.3% in the different population groups [26–30]. Moreover, schizophrenia was reported to be the common discharge diagnosis (56.1%) among mental illnesses in Ethiopia [31]. In this regard, understanding the interaction of *T*. *gondii* infection and schizophrenia will be of great value and importance. For latent *T*. *gondii* infection, the detection of anti-*T*. *gondii* IgG antibodies are considered to be a good indicator of tissue cysts in a host [32]. Herein, we examined the association between the seroprevalence of anti-*T*. *gondii* IgG and IgM antibodies and schizophrenia as compared to sex and age-matched controls.

## Materials and methods

### Study design and setting

A case-control study was conducted from February 2018 to January 2019 to determine the seropositivity and serointensity of anti-*T*. *gondii* immunoglobulins among schizophrenia patients and controls. Schizophrenia patients were recruited from Amanuel Mental Specialized Hospital (AMSH) which is the only referral psychiatric hospital in the country. AMSH is located in Addis Ketema sub-city in the capital Addis Ababa, Ethiopia. In the AMSH, more than 400 patients attend the outpatient clinic daily, and the common discharge diagnosis in the hospital was schizophrenia (56.1%) followed by bipolar disorder (20.6%), and major depression (11.4%) [31]. Sex and age-matched controls were drawn from Tikur Anbessa Specialized Hospital (TASH) which is located in the same catchment area as AMSH. TASH, which is also called Black Lion Hospital, is Ethiopia’s oldest and largest referral hospital located in the College of Health Sciences, Addis Ababa University, Ethiopia. With its more than 800 beds, TASH provides a tertiary level referral treatment and is open 24 hours for emergency services.

### Source and study populations

Patients in the AMSH and TASH were taken as the source population. Furthermore, adult schizophrenia patients in the AMSH and individuals who present for treatment of general medical conditions at TASH were taken as study populations.

### Inclusion criteria

Participation in this study was interest-based, and those who are willing and able to provide written informed consent were included. Schizophrenia cases were included based on clinical diagnosis as confirmed by clinician referral and medical record review. Controls in this study were individuals who present for treatment of general medical conditions at TASH, and who screen negative for severe psychological distress, according to the Kessler Psychological Distress Scale (K6) [33,34]. Furthermore, controls who were not taking any psychiatric medication as determined by self or medical record review and not in an inpatient until or under medical care for acute alcohol or drug intoxication were included. Controls were matched to schizophrenia cases with age and sex.

### Exclusion criteria

Study subjects who were unable to provide consent due to their unstable mental state exhibited by aggressive behaviors or patients who were unwilling to take part in the study were excluded. Controls who exhibited acute, intrusive levels of psychiatric symptoms or had acute levels of alcohol or substance abuse as demonstrated by being a current inpatient or under acute medical care were excluded. Moreover, individuals under the age of 18 years (yrs) old were excluded from the study.

### Sample size

A total of 94 study subjects (47 schizophrenia cases and 47 matched controls) were included based on the following assumptions: power 80%, confidence level (CL) 95%, odds ratio (OR) 3.24, percentage exposed among controls 37.1% [35].

### Data collection and analysis

A structured questionnaire was used to collect demographic data from study subjects. Firstly, the questionnaire was prepared in English. Then, it was translated into the Amharic language (the local language for the study site). To maintain the consistency of the content, the Amharic version of the questionnaire was translated back to English. Nurses with bachelor’s degrees took the informed consent and collected the phenotypic data from schizophrenia patients and matched controls who passed a psychological screening interview and the Kessler Psychological Distress Scale (K6). A 5 ml blood was collected from each study subject by phlebotomists from both health facilities. Shortly after blood collection, the serum was separated by centrifugation at 3500 rpm for 5 minutes and the serum was stored at -20˚C. Then, the serum samples were transported through a cold chain system using an ice bag to the International Clinical Laboratories (ICL) for the analysis of anti-*T*. *gondii* IgG and IgM antibodies through chemiluminescent immunoassay. ICL is one of the most recognized and accredited laboratories in Ethiopia which provides quality laboratory services throughout Addis Ababa and the regional cities. ICL is the first and the only laboratory in Africa accredited by Joint Commission International—USA 6 times since 2004 [36]. Test results received from ICL were interpreted as follows. Anti-*T*. *gondii* IgM test results were reported qualitatively as “Positive” and “Negative”, and the test results from ICL were used for analysis. On the other hand, anti-*T*. *gondii* IgG test results were reported from ICL quantitatively, and the following anti-*T*. *gondii* IgG reference values were used for analysis: Negative (anti-*T*. *gondii* IgG <1.6 International units per milliliter (IU/ml)), Gray zone (1.6 IU/ml < anti-*T*. *gondii* IgG< 3.0 IU/ml), Positive (anti-*T*. *gondii* IgG ≥ 3Ul/ml). For this purpose, study subjects with gray zone anti-*T*. *gondii* IgG values were considered to be negative for *T*. *gondii* infection.

### Data analysis

Data was entered and analyzed using Statistical Package for the Social Sciences (SPSS v 24). Proportions for categorical variables and mean with standard deviations (SD) for continuous variables were used as descriptive measures. The strength of associations of different factors was assessed using the odds ratio and corresponding 95% confidence interval (CI). For the multivariable analysis, variables with p-values *<*0.2 in the bivariate analysis were included. Hosmer-Lemeshow statistics were used to test the goodness-of-fit of the model. Variables, from the multivariable analysis, with p-values <0.05 were taken as statistically significant and independently associated with schizophrenia.

### Ethical consideration

Ethical approval was obtained from the Department Research and Ethical Review Committee (DRERC) of the Department of Microbiology, Immunology and Parasitology, Addis Ababa University, Ethiopia (protocol number:023/17/DMIP) and AMSH (protocol number: AM/146/4/101). Written informed consent was obtained from study participants. Confidentiality was ensured by collecting the data anonymously and coding the names of the respondents.

## Result

### Characteristics of the study subjects

A total of 94 study subjects were included in this study of which 47 (50%) were schizophrenia patients and the remaining 47 (50%) were age and sex matched controls. In both schizophrenia patients and controls, 26 (55.3%) were males and 21 (44.7%) were females. The mean ages of schizophrenia patients and controls were 29.64 ± 5.8 yrs and 30.98 ± 7.3 yrs, respectively. As compared to controls, schizophrenia patients were more likely to be illiterate (70.2% vs 44.7%; p = 0.002), unemployed (95.7% vs 57.4%; p < 0.001), unmarried (57.4% vs 17.0%; p < 0.001), and have no income (95.7% vs 57.4%, p < 0.001) (Table 1).

**Table 1:** Subject characteristics and seroprevalence of *T*. *gondii* infection among schizophrenia patients and controls.

Characteristics	Schizophrenia cases (n = 47)	Controls (n = 47)	P-value
**Sex**			
Male	26 (55.3%)	26 (55.3%)	1.000
Female	21 (44.7%)	21 (44.7%)	
**Mean age (yrs.)**	29.64 ± 5.8	30.98 ± 7.3	0.326
**Residence**			
Urban	24 (51.1%)	19 (40.4%)	0.301
Rural	23 (48.9%)	28 (59.6%)	
**Education level**			
Illiterate	33 (70.2%)	21 (44.7%)	0.002
Primary	7 (14.9%)	3 (6.4%)	
Secondary and above	7 (14.9%)	23 (48.9%)	
**Occupation**			
Employed	2 (4.3%)	20 (42.6%)	<0.001
Unemployed	45 (95.7%)	27 (57.4%)	
**Marital status**			
Not married	27 (57.4%)	8 (17.0%)	<0.001
Married	20 (42.6%)	39 (83%)	
**Religion**			
Orthodox	31 (66%)	23 (48.9%)	0.240
Muslim	10 (21.3%)	16 (34%)	
Protestant	6 (12.8%)	8 (17%)	
**Income**			
Yes	2 (4.3%)	20 (42.6%)	<0.001
No	45 (95.7%)	27 (57.4%)	
**Anti-*T*. *gondii* IgG**			
Positive	41 (87.2%)	38 (80.9%)	0.398
Negative	6 (12.8%)	9 (19.1%)	
**Mean IgG (IU/ml)**	16.5 ± 30.9	24.6 ± 44.6	0.310
**Anti-*T*. *gondii* IgM**			
Positive	0 (0%)	0 (0%)	1.000
Negative	47 (100%)	47 (100%)	
**Schizophrenia onset among anti-*T*. *gondii* IgG positives**		N/A	
Mean (SD) age of males (n = 23)	25.5 ± 5.5 yrs	N/A	0.496
Mean (SD) age of female (n = 18)	24.1 ± 7.6 yrs	N/A	
**Schizophrenia onset among anti-*T*. *gondii* IgG negatives**		N/A	
Mean (SD) age of males (n = 3)	22.0 ± 3.6 yrs	N/A	0.759
Mean (SD) age of female (n = 3)	23.7 ± 8.0 yrs	N/A	
**Mean anti-*T*. *gondii* IgG (IU/ml)**		N/A	
Schizophrenia patients with duration of illness of ≤3 ys (n = 18)	23.5 ± 44.7	N/A	0.228
Schizophrenia patients with duration of illness of >3 yrs (n = 29)	12.2 ± 17.5	N/A	

N/A: Not applicable.

### Seroprevalence of *T*. *gondii* infection

We found that 81.9% (77/94) of the total study subjects were seropositive for anti-*T*. *gondii* IgG antibody. However, none of the study participants was positive to anti-*T*. *gondii* IgM antibody. While the difference is marginal, the proportion of positive anti-*T*. *gondii* IgG antibody was higher among schizophrenic patients than controls (87.2% vs 80.9%; p = 0.398) (Table 1). We also observed a higher *T*. *gondii* infection among schizophrenia cases who are males (88.5% vs 85.7%; p = 0.779), older than 35 yrs (88.9% vs ≤88.0%; p = 0.944), and live in urban areas (91.7% vs 82.6%; p = 0.352). Yet, the observed differences are not statistically significant.

**Fig 1:**
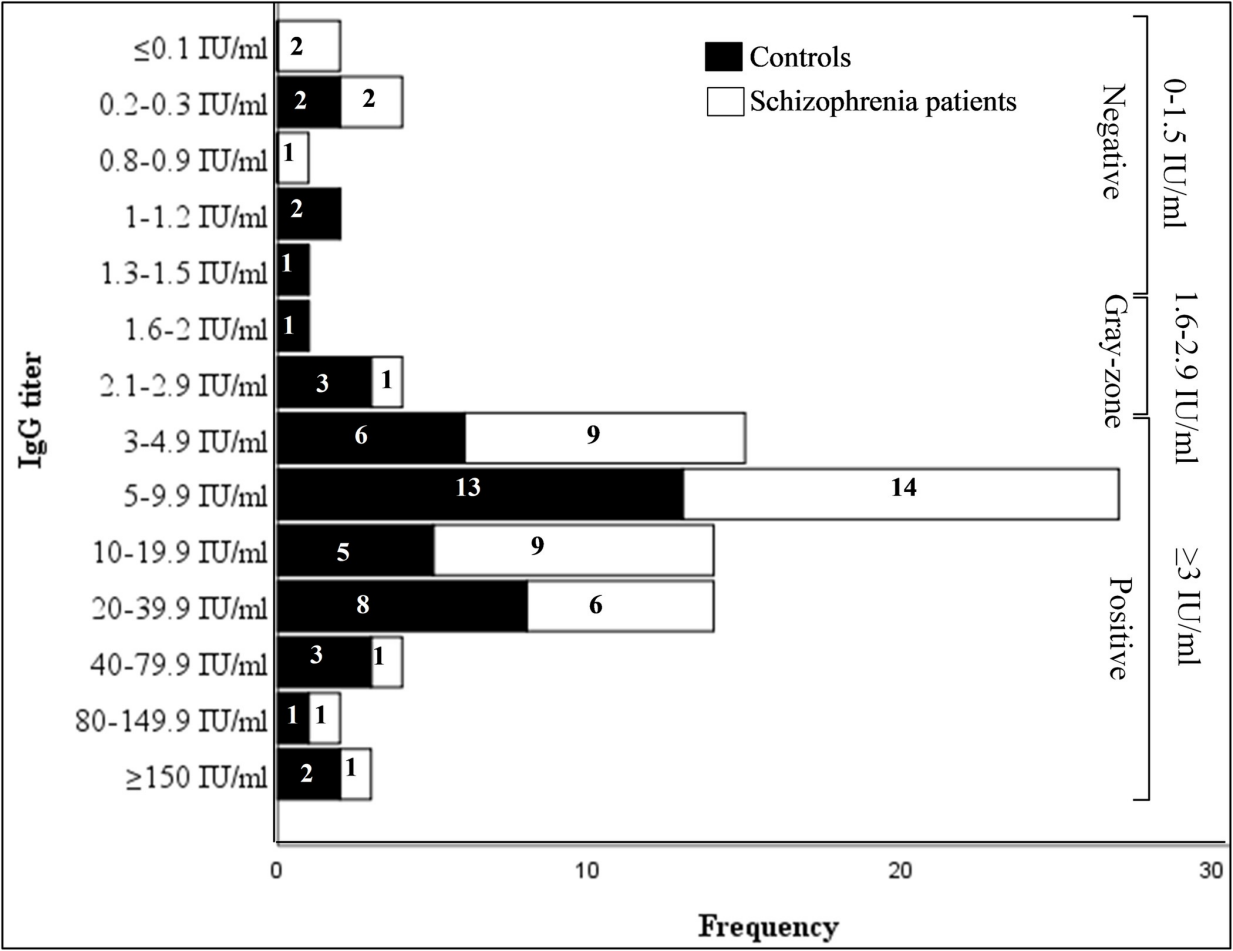
Anti-*T*. *gondii* IgG antibody titres among schizophrenia patients and controls.

We evaluated the number of positive anti-*T*. *gondii* IgG antibody titres among schizophrenia patients and controls (Fig 1). In this regard, a higher proportion of schizophrenia patients (14, 29.8%) and controls (13, 27.7%) have a positive anti-*T*. *gondii* IgG antibody titre of 5–9.9 IU/ml. However, the observed differences in the positive anti-*T*. *gondii* IgG antibody titres among schizophrenia patients and controls were not statistically significant (P = 0.58).

Furthermore, we compared the mean age of schizophrenia onset among *Toxoplasma* positive and *Toxoplasma* negative subjects (Table 1). Schizophrenic cases with *T*. *gondii* infection have a higher mean age of schizophrenia onset as compared to cases who were *Toxoplasma*-negative (24.9±1.0 yrs vs 22.8±2.3 yrs; p = 0.463). Likewise, in both males and females, the mean age of schizophrenia onset was higher among cases with *T*. *gondii* infection (25.5 ± 5.5 yrs vs 22.0 ± 3.6 yrs in men and 24.1 ± 7.6 yrs vs 23.7 ± 8.0 yrs in women). We also determined the correlation between anti-*T*. *gondii* IgG antibody and duration of illness among schizophrenic cases (Table 2). Although the relationship is weak, a decrease in anti-*T*. *gondii* IgG antibody was observed with an increase in the duration of illness (Pearson Correlation coefficient (*r*) = 0.041; p = 0.787). Particularly, the proportion of *T*. *gondii* infection was significantly higher among schizophrenic cases who had the disease for not more than three yrs (100.0% vs 79.3%; p = 0.039). Likewise, although the difference is statistically insignificant, the mean anti-*T*. *gondii* IgG antibody was higher among schizophrenic cases who had the disease for less than three yrs (23.5 ± 44.7 IU/ml vs 12.2 ± 17.5 IU/ml; p = 0.23).

**Table 2:** Factors associated with *T*. *gondii* infection among schizophrenia cases and controls.

Variables	*T*. *gondii* IgG among Schizophrenia cases			*T*. *gondii* IgG among Controls		
	Negative	Positive	P-value	Negative	Positive	P-value
**Duration of illness in schizophrenia**						
≤3 ys (n = 18)	0 (0.0%)	18 (100.0%)	0.039	N/A	N/A	N/A
>3 yrs (n = 29)	6 (20.7%)	23 (79.3%)		N/A	N/A	
**Income**						
Yes	0 (0.0%)	2 (100.0%)	0.580	7 (35.0%)	13 (65.0%)	0.017
No	6 (13.3%)	39 (86.7%)		2 (7.4%)	25 (92.6%)	
**Addiction to khat and alcohol**						
Yes	0 (0.0%)	10 (100.0%)	0.173	1 (20.0%)	4 (80.0%)	0.959
No	6 (16.2%)	31 (83.8%)		8 (19.0%)	34 (81.0%)	
**Floor at home**						
Soil	4 (9.1%)	40 (90.9%)	0.004	8 (18.6%)	35 (81.4%)	0.756
Cement/ceramic	2 (66.7%)	1 (33.3%)		1 (25.0%)	3 (75.0%)	
**Contact with cats and dogs**						
Yes	4 (11.1%)	32 (88.9%)	0.539	7 (22.6%)	24 (77.4%)	0.405
No	2 (18.2%)	9 (81.8%)		2 (12.5%)	14 (87.5%)	
**Eat raw meat**						
Yes	6 (13.0%)	40 (87.0%)	0.699	9 (20.0%)	36 (80.0%)	0.482
No	0 (0.0%)	1 (100.0%)		0 (0.0%)	2 (100.0%)	
**Eat chicken egg**						
Yes	6 (13.3%)	39 (86.7%)	0.580	9 (20.9%)	34 (79.1%)	0.309
No	0 (0.0%)	2 (100.0%)		0 (0.0%)	4 (100.0%)	
**Rat and cockroaches at home**						
Yes	0 (0.0%)	1 (100.0%)	0.699	0 (0.0%)	2 (100.0%)	0.482
No	6 (13.0%)	40 (87.0%)		9 (20.0%)	36 (80.0%)	
**Access to treated water**						
Yes	6 (13.0%)	40 (87.0%)	0.699	9 (20.0%)	36 (80.0%)	0.482
No	0 (0.0%)	1 (100.0%)		0 (0.0%)	2 (100.0%)	
**Eat raw vegetables**						
Yes	4 (14.8%)	23 (85.2%)	0.625	5 (21.7%)	18 (78.3%)	0.659
No	2 (10.0%)	18 (90.0%)		4 (16.7%)	20 (83.3%)	
**Drink unpasteurized milk**						
Yes	6 (13.3%)	39 (86.7%)	0.580	9 (20.9%)	34 (79.1%)	0.309
No	0 (0.0%)	2 (100.0%)		0 (0.0%)	4 (100.0%)	

N/A: Not applicable.

### Factors associated with *T*. *gondii* infection

Primarily, we assessed factors associated with *T*. *gondii* infection among schizophrenic cases (Table 2). Based on our findings, *T*. *gondii* infection is significantly higher among cases who live in homes with soil floors as compared to those who live in homes with cement/ceramic floors (90.9% vs 33.3%; p = 0.004). Furthermore, although the differences are not statistically significant, we observed a marginally higher proportion of *T*. *gondii* infection among schizophrenia cases who had direct contact with cats and dogs (88.9% vs 81.8%; p = 0.539), addicted to Khat (a stimulant plant leaf) and alcohol (100.0% vs 83.8%; p = 0.173), and no access to safe water (100.0% vs 87.0%; p = 0.699).

Then, we compared the differences in *Toxoplasma*-associated factors among cases and controls who are seropositive for anti-*T*. *gondii* IgG antibodies. For this purpose, we screened study subjects who had *T*. *gondii* infection and we made a multivariate analysis on variables with p-values < 0.2 in the bivariate analysis (Table 3). Among all study subjects who have *T*. *gondii* infection, subjects who are addicted to khat and alcohol are about seven times more likely to have schizophrenia (71.4% vs 47.7%, OR = 7.13, p = 0.024). Moreover, unmarried subjects with *T*. *gondii* infection are about six times more likely to have schizophrenia (76.7% vs 36.7%, OR = 5.53, p = 0.009). While the difference approached but did not reach significance (p = 0.075), *T*. *gondii* infected subjects who have contact with cats and dogs are about three times more likely to have schizophrenia (57.1% vs 39.1%, OR = 3.26).

**Table 3:** Multivariate analysis of factors associated with schizophrenia cases and controls who tested positive for anti-*T*. *gondii* IgG antibodies.

	Schizophrenia			
	Yes (n = 41)	No (n = 38)	OR (95% CI)	P-value
**Marital status**				
Not married	23 (76.7%)	7 (23.3%)	1.00	
Married	18 (36.7%)	31 (63.3%)	5.53 (1.54, 19.85)	0.009
**Addiction to khat and alcohol**				
Yes	10 (71.4%)	4 (28.6%)	1.00	
No	31 (47.7%)	34 (52.3%)	7.13 (1.30, 39.04)	0.024
**Contact with cats and dogs**				
Yes	32 (57.1%)	24 (42.9%)	1.00	
No	9 (39.1%)	14 (60.9%)	3.26 (0.89, 11.91)	0.075

## Discussion

In this study, the prevalence of *T*. *gondii* infection (IgG+/IgM-) among schizophrenia patients was 87.2%. Our finding was consistent with a similar study in Lebanon in which a high (79%) seroprevalence of *T*. *gondii* infection was reported [37]. However, our finding was higher than previous studies in Gondar, Ethiopia 33.6% [38], Mashhad, Iran 40.12% [39], and New Zealand 33.33% [40]. The differences in the prevalence of *T*. *gondii* infection among schizophrenia patients might be attributed to either a geographic variation that influences the prevalence of toxoplasmosis or methodological differences among studies.

Schizophrenic cases, in this study, had a marginally higher seroprevalence of *T*. *gondii* infection as compared to controls (87.2% vs 80.9%). However, the difference was not statistically significant. Indeed, most available reports (reviewed in [10]) suggests a strong association between *T*. *gondii* infection and altered mental status including schizophrenia. Schizophrenia is a chronic disease of the central nervous system (CNS), and neurotropic infectious agents such as *T*. *gondii* have been associated with its occurrence [14]. Yet, the causal relationship between *T*. *gondii* infection and schizophrenia is not well defined. The effect of *T*. *gondii* infection on mental disorders remains ambiguous, and reports vary considerably between studies. Previous studies from Ethiopia [38], Nigeria [41], Libya [42], and China [43] showed a significantly higher *T*. *gondii* infection among schizophrenic cases. However, we didn’t detect a significant difference in the seroprevalence of anti-*T*. *gondii* antibodies among controls and schizophrenia cases despite the observed high *T*. *gondii* infection in the later. This might be attributed partly to either the small sample size of the study, or the inclusion of inpatient schizophrenic cases who had been under antipsychotic medications. Available reports suggest that the replication of *T*. *gondii* will be inhibited by antipsychotic drugs used in the management of mental disorders [44–46] in addition to their roles in either decreasing the dopamine concentration or down-regulating the activity of its receptors on neural cells [47]. In line with this, possibly due to the extended therapeutic effects of antipsychotic drugs, we found a significantly lower *T*. *gondii* infection among schizophrenic cases who were taking antipsychotic medication for more than three yrs (79.3% vs 100.0%, p = 0.039). In addition, the lack of significant difference in the *T*. *gondii* infection between schizophrenia cases and controls in our case might perhaps be due to the high burden of *T*. *gondii* infection in the general population of Ethiopia. In areas with low prevalence of *T*. *gondii* such as China (5.13%) [48] and Durango City, Mexico (6.1%) [49], *T*. *gondii* infection was shown to have a positive association with schizophrenia [50–52]. While this may hold true in a general sense, Xiao and colleagues from China reported no correlation between *T*. *gondii* infection and psychiatric disorders despite low *T*. *gondii* prevalence in the general population [48,53]. Certainly, there are emerging reports with no evidence of associations between schizophrenia and toxoplasmosis. Sugden and colleagues, in a population-representative birth-cohort, found no significant association between *T*. *gondii* seropositivity and schizophrenia [40]. El Mouhawass et al [37] also demonstrated no significant difference between schizophrenia cases and controls for anti-*T*. *gondii* IgM−/IgG+ antibodies. Likewise, studies from Iran [39] and Germany [54] also reported that *T*. *gondii* seropositivity was not significantly associated to schizophrenia cases. Thus, further studies are needed to narrow the observed knowledge gaps.

Schizophrenia symptoms usually manifest late in adolescence or young adulthood [55]. However, whether *T*. *gondii* infection preceded the onset of schizophrenia is not clearly understood. In this regard, we observed that schizophrenia cases with a positive anti-*T*. *gondii* IgG antibodies had a higher mean age of schizophrenia onset (25.5 ± 5.5 yrs among males and 24.1 ± 7.6 yrs among females) as compared to schizophrenia cases with a negative anti-*T*. *gondii* IgG antibody (22.0 ± 3.6 yrs among males and 23.7 ± 8.0 yrs among females). Consistent with our finding, the study in the Czech Republic showed that the mean age of schizophrenia onset was higher in *Toxoplasma*-infected subjects as compared to *Toxoplasma*-free schizophrenia cases [56]. While our results are not conclusive, owing to the higher mean age of schizophrenia patients with a positive anti-*T*. *gondii* IgG antibody, *T*. *gondii* infection might have preceded the onset of schizophrenia. In line with this, a piece of strong evidence from the prospective study by Niebuhr et al [5] showed that *T*. *gondii* infection precedes the onset of schizophrenia by 6–36 months.

Schizophrenia is caused by many environmental and genetic factors, which are both additive and interchangeable in their effects [57]. Our findings show that schizophrenia cases with *T*. *gondii* infection were about seven times more likely to have addictions to khat and alcohol as compared to controls with toxoplasmosis (p = 0.024). This may strengthen the assumption that *T*. *gondii* infection is an important cause of schizophrenia in subjects with other environmental and genetic predispositions. Cats are a definitive host for *T*. *gondii*, and play an important role in disease transmission. In Ethiopia, cats being the closest living animals are linked to the reportedly high seroprevalence of *T*. *gondii* infection [58,59]. Patients who have schizophrenia or other mental disorders were shown to have greater exposure to cats [60]. Accordingly, in this study, schizophrenia patients with *T*. *gondii* infection were about three times more likely to have contact with cats and dogs (p = 0.075). Humans may become infected by contact with cat feces which shed the infective oocyst of *T*. *gondii*. Oocysts can survive and remain infective for about two years in soil [61]. In this regard, contact with soil could be a potential risk factor for *T*. *gondii* infection [62,63]. Likewise, schizophrenic cases who live in homes with soil floors in this study had a significantly higher *T*. *gondii* infection as compared to those who live in homes with cement/ceramic floors (p = 0.004).

This study is not without limitations. On the one hand, we were limited to a total of 94 study subjects due to budget constraints. In this regard, our failure to find a significant association between schizophrenia and toxoplasmosis despite the higher prevalence of *T*. *gondii* infection among cases might be due to the small sample size of the study. On the other hand, we recruited study controls from the referral hospital which is in the same catchment area as the mental hospital where cases were recruited. However, we believe that controls drawn from the community who are the nearest neighbours to the schizophrenic cases would provide better information on this.

## Conclusion

We didn’t observe a significant difference in the seroprevalence of *T*. *gondii* infection among schizophrenia patients and controls. However, our data showed that addiction to khat and alcohol is a risk factor for schizophrenia among study subjects with *T*. *gondii* infection. We believe that future community-based studies should consider the inclusion of treatment naïve schizophrenic cases which will allow determining the possible therapeutic effect of antipsychotic drugs on *T*. *gondii* mediated mental disorders including schizophrenia.

## Abbreviations

ICL: International Clinical Laboratories
IgG: Immunoglobulin G
IgM: Immunoglobulin G
IU/ml: International units per milliliter
OR: Odds ratio
Yrs: Years wrote the first draft and the revised version of the manuscript
